# Structures promoting research, training, and technology transfer in mobility: lessons learned from a visit to European centers

**DOI:** 10.1186/1743-0003-9-19

**Published:** 2012-03-30

**Authors:** Michael L Boninger, Rachel E Cowan, Benjamin J Fregly

**Affiliations:** 1 Department of Physical Medicine & Rehabilitation University of Pittsburgh School of Medicine 3741 5th Avenue, Suite 201 Pittsburgh, PA 15260; 2 Human Engineering Research Laboratories VA Pittsburgh Healthcare System 6425 Penn Avenue, Suite 400 Pittsburgh, PA 15206; 3 Department of Neurological Surgery The Miami Project to Cure Paralysis University of Miami Miller School of Medicine 1095 NE 14th Terrace Miami, FL 33136; 4 Department of Mechanical and Aerospace Engineering 231 MAE-A Building, P.O. Box 116250 University of Florida Gainesville, FL 32611

## Abstract

The purpose of this paper is to describe the education, research, technology transfer, and cooperative models that appear to have the greatest likelihood of successfully tackling the issue of technology to improve mobility. Ideally better models in each of these areas will lead to an increased number of researchers who are more productive. There will be increased international collaboration that will allow for better research with small and/or disadvantaged populations, and the research completed will lead to changes in clinical care that positively impact individuals with impair mobility.

## Introduction

Over 40 million Americans live with a disability. According to the 2007 IOM report on disability, disability in the form of limited activities and restricted participation in social life is not an unavoidable result of injury and chronic disease [1]. It results, in part, from choices society makes about working conditions, health care, transportation, housing, and other aspects of our environment. This is a powerful statement that places the cause of disability on society. According to the ICF model [2], disability results form an interaction between an individual and their environment. Technology for mobility can be a very important mediator of this interaction. A simple example is the well accepted assistive technology for mobility, the wheelchair. In its simplest form, the wheelchair enables an individual who cannot walk to travel from point A to point B. For the task of going from point A to B in an airport, this technology is nearly perfect. However, for getting on the airplane, it fails. The chair will not fit down the aisle and a transfer is needed to get out of the chair and into an airplane seat.

Could additional technology solve this problem in a way that is seamless to the wheelchair user? The answer is undoubtedly yes; however, the technology that enables an individual with lower limb paralysis to transfer into an airplane seat does not exist. One could easily argue that with existing materials a device could easily be fabricated, so why don't we have such a device? One answer is that society has not chosen to invest the money needed to provide true accessibility. While this answer is true, the other truth is that groups are needed that have the will and expertise to focus on fixing this and the myriad of problems faced by individuals with mobility impairment. At present there is a shortage of well trained researchers working on these problems [3]. This shortage has been recognized by major research funding agencies in the United States who have set up various funding mechanisms to promote research in the critical area.

As part of a National Science Foundation (NSF) sponsored trip, a group of primarily senior scientists traveled to a number of the best rehabilitation research laboratories in Western Europe. The purpose of the trip was to explore cutting edge research in the area of technology to improve mobility. Other papers in this series explore specific technology. The purpose of this paper is to describe the *education*, *research*, *technology transfer*, and *cooperative models *seen on the trip that appear to have the greatest likelihood of successfully tackling the issue of technology to improve mobility. Ideally better models in each of these areas will lead to an increased number of researchers who are more productive. There will be increased international collaboration that will allow for better research with small and/or disadvantaged populations. And the research completed will lead to changes in clinical care that positively impact individuals with impair mobility. Below we discuss the four areas listed above and provide examples of what we believe to be best structures.

## Methods

A multidisciplinary team of primarily senior researchers was assembled by the NSF working in conjunction with the World Technology Evaluation Center (WTEC, http://www.wtec.org/accessed 3/12/2012). The team consisted of two scientists working in mobility technology who had mobility impairments and were consumers of technology, two physicians specializing in Physical Medicine and Rehabilitation, five individuals with engineering degrees, one physical therapist, and one exercise physiologist with a PhD in Rehabilitation Sciences. The team came from major universities across the United States and also contained two individuals working for funding agencies. The team worked to identify European laboratories that they felt were leaders in the field and also spanned a variety of rehabilitation technology approaches. Based on the team's findings, the WTEC organization put together an itinerary for laboratory site visits. The trip was limited to one week and therefore multiple outstanding laboratories were likely not included. In addition to visiting universities, the team also visited private companies and funding agencies.

The team gave each site a questionnaire to complete prior to the trip, and answers to the questionnaire are part of the information used to present the results below. The team broke up into two groups that traveled separately. At each location the purpose of the visit was presented. The trip covered 8 countries and approximately 30 labs. Presented below are summary findings with illustrative examples of what the team believes worked well from education, research, technology transfer, and across-country collaboration perspectives. In addition, critical gaps are identified.

## What Worked

### Education

To advance the field of mobility related research, structures are needed to support the creation of the next generation of scientist working in this area. A common theme across multiple programs that address more than just education is the need for multidisciplinary teams. The best educational programs embraced a multidisciplinary approach during schooling, with students studying in different areas working as a team. This approach also leads to multidisciplinary mentorship. An example of an innovative approach to interdisciplinary education is provided by the Imperial College in London. At this institution there is a course entitled REHANDFUN. In this course a multidisciplinary group of students from biology, engineering, and computer science receive a kit and are instructed to develop a therapeutic game. The intent of the course is to develop student knowledge of rehabilitation technology, human center design, and teamwork. The Arts Et Metiers Paristech provides another example. The school works with Paris Descartes Medical University in an international master's degree program in Biomedical Engineering. In this program half of the students admitted have an engineering background and half have a clinical background. This combination assures clinical and engineering representation throughout the coursework.

Other innovations in education include specialized course work. One recurrent theme was the growing movement away from didactic lessons to more experiential based learning. The REHANDFUN course mentioned above represents such a move. This concept has been fully embraced by Aalborg University, which from its beginning has used problem-based learning as roughly 50% of its curriculum. This approach is encapsulated by the following saying:

**Tell me and I will forget**,

**Show me and I will remember**,

**Involve me and I will understand**,

**Step back and I will act**.

Old proverb

An observation by our group involved proximity. It is clear that in the educational setting, interdisciplinary teams work best when in close proximity to each other. Many institutions talked about interdisciplinary work, but in the absence of proximity, the collaboration often seemed to be less effective.

A major gap in the educational area--a gap that is thematic to all areas--involved a lack of individuals with mobility impairment among the student or teaching populations. While interdisciplinary teams can help, having direct involvement of technology users is critical and was largely lacking from the majority of the educational institutions visited. One notable exception was a master's student with a mobility impairment working on the haptic trainer at the IPK Fraunhofer in Berlin. This issue is not the same as involving consumers as consumers, which is also an important part of the process. Rather, what is missing is students and teachers with disability.

### Research

**Figure 1:**
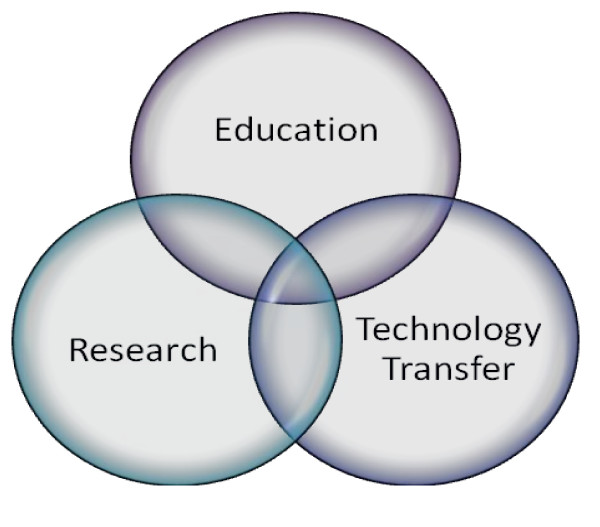
**Overlapping realms needed for success**.

Research and education go hand in hand even in industrial or hospital settings. Thus the separate sections of this paper are really overlapping (see Figure 1). Given this overlap, it is not surprising that multidisciplinary teams are also critical to the research mission. In some hospital settings, we met with clinicians trying to solve relatively simple engineering problems, while at some engineering schools, we met engineers developing products with questionable clinical applicability. One key part of the interdisciplinary team at a number of institutions was an industry partner. The technology transfer circle in the figure above can be represented as an industry partner.

A unique example of a multidisciplinary approach is provided by the Technology Research for Independent Living (TRIL) in Ireland http://www.trilcentre.org/who-we-are.htmlaccessed on 3/8/2011. The center is unique in that the multidisciplinary team extends beyond engineers and clinicians. TRIL focuses on enabling technology development and evaluation to support independent living. This center includes ethnographers and designers as part of the team. Ethnographers spend extended time understanding the day-to-day lives of end users. The design-ethnography process facilitates the development of research prototypes leading to execution of experiments with older people in their own homes.

Another important aspect of a successful program is infrastructure. Many of the research tools related to movement are expensive. These tools include kinetic and kinematic measurement devices, virtual reality, and robotic and clinical devices, all with a large price tag. Under-resourced or isolated investigators may simply not have the needed research apparatus to make an impact, even if their ideas are valuable. The resources can come from a single institution like the University of Twente (see below) or from collaborative partnerships as in Zurich. In Zurich there were a number of institutions, many of which seemed to work together well. These included ETH Zurich http://www.ethz.ch/index_EN accessed on 3/11/2011, Balgrist Hospital, which is affiliated with the University of Zurich http://www.balgrist.ch/desktopdefault.aspx accessed 3/11/2011, the Centre for outpatient Rehabilitation (ZAR) in Zurich, and Hocoma. These institutions are in close proximity to one another and have many projects that appear to overlap. The industry partner, Hocoma, provides one outlet for technology transfer, and the combined resources are impressive.

Similar to what was reported above, a substantial gap exists in inclusion of end users of mobility technology on the research team. We observed very few end users involved as researchers and, remarkably, many of the research laboratories devoted to mobility related research were only marginally accessible to an individual in a wheelchair. Another gap identified by the team was related to replication of work. Many laboratories seemed to be pursuing similar research ideas, and some were repeating the work of others or work done years ago. While to some extent this situation is unavoidable and even positive at times, the amount of overlap appeared to be too much. This redundancy was most noticeable in smaller programs that had not yet reached a critical research mass.

### Technology Transfer

The ultimate test of success is transfer of research to commercially viable and clinically useful products. This goal continues to be problematic in the technology for mobility area and the cause is multi-factorial. One factor already discussed is non-inclusion of end users in the process or as part of the team. Another factor is likely regulatory hurdles and commercial expenses related to bringing medical products to market. One successful model we observed was having a built-in industry collaboration. At some locations this collaboration was achieved by having a small startup company nearby that began because of a specific technology. Such an example is the collaboration between Smartex, a company that focuses on wearable fabric based sensors, and the University of Pisa. The company and a portion of the University are located on two adjacent floors of the same building in an industrial park. While they remain separate financial entities in separate office spaces, there is obvious cross fertilization that impacts the technology transfer process, with researchers from both entities flowing freely between the two office spaces.

A large scale example is provided by the University of Twente and its affiliated programs. MIRA (Institute for Biomedical Technology and Technical Medicine), one of the affiliate programs, has government support, cuts across all departments, and assists in bringing technology to market. There are 250 researchers employed by MIRA and there have been 8 spinoff companies like Xsens http://www.utwente.nl/mira/entrepreneurship/miras_spin_offs/accessed 3/13/2011. Xsens makes motion capture systems used for various purposes by researchers and product developers in movement science. Researchers may obtain a stake in spinoff companies, which are primarily funded by outside investors. MIRA's role is helping to bring research to a marketable point and connecting investors with researchers. Specific knowledge related to regulatory hurdles is one of the advantages MIRA and other University of Twente related organizations bring to the task of technology transfer.

In general, larger, better supported, more multidisciplinary organizations that included industrial partners led to more successful technology transfer. In a number of sites the government was providing clear financial support to educational institutions and companies as a means of assisting with the technology transfer process. It is clear that countries that don't support technology transfer are putting the researchers in their country at a disadvantage. However, at more than one location, there was a sense that the government funding sometimes continued despite a lack of success in commercializing a product.

Another concern related to technology transfer and the models seen was in the area of conflict of interest. It was clear that a number of investigators had an interest in companies working to commercialize their products. The mechanisms for dealing with the conflicts of interest appeared to be less defined then what is in place in the United States. Overly restrictive conflict of interest policies can likely stifle technology transfer, whereas the absence of policies could lead to conflict of interest causing bad science. This area is one that clearly needs a measured approach.

### Cooperation Between Countries

**Figure 2:**
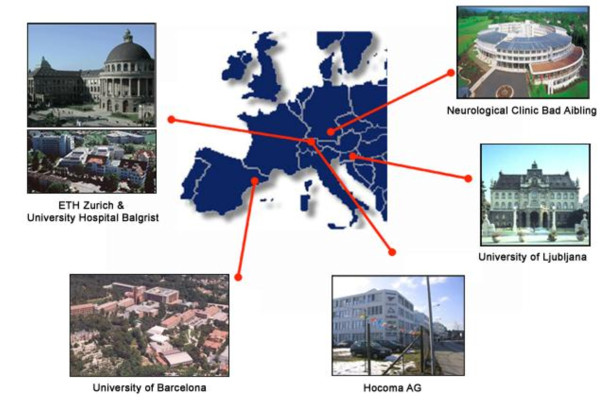
**Example of multi-country collaborative effort**.

It is widely acknowledged that cooperation across research groups can facilitate scientific gains. Collaborators across counties can bring unique expertise, knowledge of different cultures, and help with recruitment. Recruitment help is especially important in conditions that impact relatively small populations such as spinal cord injury. The environment in Europe is heavily influenced by European Union (EU) funding, a portion of which requires inter-country collaboration. An example of this collaboration is provided by MIMICS (Multimodal Immersive Motion rehabilitation with Interactive Cognitive Systems, http://www.mimics.ethz.ch/index.php?page_id=0accessed3/12/2012). The main hypothesis of this project is that movement training for neurorehabilitation can be substantially improved through immersive and multimodal sensory feedback. Figure 2 shows the sites involved. The collaboration is expected to end in a commercial product and the pathway from technical development through clinical evaluation and commercialization was well defined as part of the proposal.

It was clear from our visit that collaborations fostered by requirements imposed by funding mechanisms lead to positive research outcomes. It was also clear that not all collaborations were functioning in a positive fashion and that the drive for funding may bring researchers together on paper more than in reality. We observed difficulties in collaboration when investigators were separated by a few kilometers, let alone by great distances. Having stated these potential pitfalls, the forced collaboration model appears to have many more advantages then disadvantages.

## Recommendations

Based on the trip and subsequent meetings, a number of recommendations can be made.

1) Institutions should work to establish multidisciplinary teams in both research and educational settings.

This recommendation is not new or particularly innovative. The trip reinforced this need. It is clear that the teams must include clinicians, engineers, industrial partners, and consumers of the technology. These teams must follow an interactive design process in which all parties are active. It is also clear that proximity is required for these teams to work together effectively. The ideal situation is for all team members to work in the same building. We observed that even a long walk across campus could deter the type of collaboration that leads to success.

2) Technology transfer issues should be supported by policy.

Technology transfer can be the point at which good ideas fail. Including industry in a multidisciplinary team can greatly facilitate the technology transfer process. However, this approach brings up issues related to intellectual property and conflict of interest. On this trip, technology transfer was facilitated when university, industry, and government policies were aligned. Policies that allow ownership for faculty seemed to facilitate faculty involvement in technology transfer. Clear policy allows for early discussions on process that could avoid conflict.

3) Educational programs should incorporate experiential learning.

Experiential learning in a multidisciplinary team has a number of benefits that involve much more than absorbing the material. Provided the team has the key members noted in recommendation [1] above, students will learn how to interact with different disciplines and with individuals with disabilities. The way each discipline speaks and thinks will become apparent. The final product is likely to be better, as are the students.

4) Universities, governments, and industry need to invest to create centers

Creating technology to assist with mobility is expensive. Substantial infrastructure is needed to foster success. Thus, a center of excellence model is needed to achieve major gains. The center of excellence model requires funding, and more centers are needed to tackle the major problems facing individuals with mobility impairments.

5) Targeted programs are need to encourage individuals with disabilities to enter engineering

The trip furthered amplified the need for individuals with disabilities to be completely integrated into development of assistive technologies. The ideal situation is when they contribute clinical, engineering, and/or design knowledge along with their knowledge of living with a mobility impairment. Getting individuals with disabilities to pursue careers in these fields will require a focused effort and is worthy of investment.

## Conclusions

The trip provided numerous examples of what worked when promoting education, research, and technology transfer in assistive technology to improve mobility. Funding agencies have the ability to force change. It is not possible to state if the models we observed on our trip are worse or better than those present in the US. What is clear is that there is much to be learned from this type of trip. Information exchange in both directions will help both the European Union and the US. Future trips should include other parts of the world.

## Competing interests

The authors declare that they have no competing interests.

## Authors' contributions

All authors (MB, RC, BF) contributed equally to the conception and to writing the manuscript, and have read and approved the final manuscript.
